# The relationship between the Dark Triad and bullying among Chinese adolescents: the role of social exclusion and sense of control

**DOI:** 10.3389/fpsyg.2023.1173860

**Published:** 2023-07-10

**Authors:** Yongqi Huang, Xiong Gan, Xin Jin, Shijie Rao, Binbin Guo, Zijian He, Zixu Wei

**Affiliations:** Department of Psychology, College of Education and Sports Sciences, Yangtze University, Jingzhou, China

**Keywords:** Dark Triad, bullying, social exclusion, sense of control, adolescent

## Abstract

**Introduction:**

Abundant evidence has proved the association between the Dark Triad and bullying. However, the underlying mechanisms of this relationship are still not fully understood. Based on the temporal need-threat model, three studies were designed to explore the mediating role of social exclusion and sense of control in this research.

**Methods:**

In study 1 we recruited 571 Chinese adolescents (*M*_age_ = 14.53, *SD* = 0.716) to participate in a cross-sectional study. And two experiments were respectively designed in Study 2 (*N* = 88) and Study 3 (*N* = 102) to verify the effects of real and cyber social exclusion on adolescent bullying behavior.

**Results:**

Study 1 showed that social exclusion and sense of control would play the serial mediating role in the relationship between the Dark Triad and bullying (except social exclusion as a mediator between the Dark Triad and cyberbullying). Study 2 and 3 showed that adolescents with high Dark Triad show lower sense of control and more bullying behavior after experiencing social exclusion.

**Discussion:**

These findings extend the research on the Dark Triad and bullying by providing a solid empirical foundation and intervention strategies to avoid bullying so that the problem can be rationally and scientifically approached.

## 1. Introduction

Bullying is a widespread public health issue among children and adolescents, it is usually defined as the intentional, repeated and sustained negative behaviors of the bullied by one or more peers (Olweus, 1978). More noteworthy is the power imbalance between the perpetrator and the target (Olweus, 1993). School bullying is frequently observed in the traditional forms (physical, verbal, relational), and cyberbullying. Different from traditional bullying, cyberbullying is a form of bullying wherein the perpetrator disseminates offensive information through digital media with the intention of harming or discomforting others (Smith et al., 2008; Tokunaga, 2010). It can be generated both directly and indirectly (Hong et al., 2018). Numerous studies have identified three distinct groups of children who are involved in bullying: the bullies, the victims and those who both bully others and are also bullied (the bully/victims) (Smith et al., 1993; Salmivalli et al., 1996). For cyberbullying, research has shown that students often differ in the methods of cyberbullying but not in their roles (bully, victim, witness) (Law et al., 2012). In China, a social survey showed that 59.4% of the 2002 respondents had experienced or witnessed incidents of school bullying, and the percentage was significantly higher for boys (66.0%) than for girls (52.0%) (China Youth Daily, 2018). In 2021, the General Office of the Ministry of Education of China issued the *Work Plan for the Prevention of Bullying among Primary and Middle School Students*, stating that we should continue to do a good job in preventing and controlling bullying among primary and middle school students (Ministry of Education of the People's Republic of China, 2021). The harmful effects of bullying in schools on the physical and mental growth of teenagers cannot be ignored. Either form of bullying can result in psychological or physical discomfort or harm to the victim, such as truancy, reduced self-esteem, academic performance and self-confidence, depression, anxiety and insomnia (Raskauskas and Juliana, 2009; Tokunaga, 2010; Cassidy et al., 2013). Furthermore, a recent study shows that kids who are bullied in childhood have a higher risk of suicide in adulthood (Geoffroy et al., 2022). Thus, it is essential to investigate which factors facilitate or curb bullying in order to implement interventions to alleviate individuals’ bullying behavior.

### 1.1. Dark Triad and bullying

There are many factors that affect school bullying, of which personality traits do have a noticeable effect (Veenstra et al., 2005; Mitsopoulou and Giovazolias, 2015). One of these is the Dark Triad, which describes a cluster of antisocial personality traits consisting of Machiavellianism, narcissism, and psychopathy (Paulhus and Williams, 2002). Machiavellianism describes the psychological and behavioral characteristics of manipulation, pursuit of self-interest, and deception (Christie and Geis, 1970). Narcissism is characterized by a propensity towards entitlement, egocentrism, exhibitionism, and grandiosity (Miller et al., 2011). Psychopathy embodies antisociality, heartlessness, and impulsivity (Lilienfeld, 2018). A latest longitudinal study shows that psychopathy and Machiavellianism were found to share significantly stronger associations with bullying in comparison to the relationships between narcissism and bullying at each time point (Davis et al., 2022). Thus, it is not surprising that research has revealed that the Dark Triad traits are positively related to bullying and cyberbullying perpetration among adolescents and young adults (Andreou, 2004; Fan et al., 2016; van Geel et al., 2016, 2017). However, the study in this field has not yet been thoroughly examined to investigate where and how to affect young people bullying behavior. It is essential to investigate potential risk factors and mechanisms of adolescent bullying to develop more efficient prevention programs.

Research has found that rejection and social exclusion are possible factors in the occurrence of bullying (Dorte, 2012; Hinduja and Patchin, 2022), with almost all perpetrators of school shootings in the USA experiencing rejection or exclusion by peers (Leary et al., 2003). The temporal need-threat model (Williams, 2009) suggests that individuals face a depletion of resources if they suffer chronic social exclusion or fail to meet impaired basic needs. To seek satisfaction or compensation for needs, individuals often respond with aggression. This means that the excluded individual compensates for the compromised basic needs by regaining sense of control or dominance over the relationship through aggression. This is where exclusion becomes a potential threat to society. Social exclusion can lead to reduced self-control (Leary et al., 2006), blocked sense of control (Williams, 2009) and so on. It has been found that when the excluded lose their sense of control, they become more aggressive (Warburton et al., 2006). Besides, aggressive behavior can also lead to peer rejection. For example, in school, children who exhibit aggressive behavior are more likely to be rejected and isolated by their peers (Beeson et al., 2020). This may be because the aggressive behavior of these children causes discomfort and fear among their peers, making them more willing to keep their distance and resulting in exclusionary behavior. Social exclusion is common in our life (Nezlek et al., 2015). People with certain personality traits which we think are unpopular with others (e.g., the Dark Triad) are particularly vulnerable to rejection and exclusion (Baumeister and Tice, 1990). Meanwhile, the use of indirect violence against others, such as peer rejection and social exclusion, is also more likely to occur among these kids and adults (Heym et al., 2019; Davis and Vaillancourt, 2022). Due to the bidirectional relationship between exclusion and attack, they may mutually promote each other, forming a vicious cycle. Based on our research content, this study mainly focuses on the motivation process of Dark Triad adolescents in generating school bullying. We can therefore assume that social exclusion is a particularly serious risk factor for the Dark Triad.

In order to optimize the efficacy of interventions to counteract the detrimental impacts of social exclusion, our goal in the current study is to explore the mediators that underlie this association. Specifically, we examined whether social exclusion and sense of control mediate the relationship between the Dark Triad and adolescent school bullying using the temporal need-threat model.

### 1.2. The mediating role of social exclusion

However, the relationship between the Dark Triad and school bullying seems to be mediated by other variables (Davis et al., 2022). Social exclusion refers to interpersonal interactions in which individuals are rejected and expelled due to a failure to make a sufficient and necessary contribution to the group or they carry certain personality traits that are not welcomed by others (Baumeister and Tice, 1990). According to the temporal need-threat model, the negative impact of social exclusion on individuals consists of reflexive stage, reflective stage, and resignation stage. In the reflective stage, the individual adopts certain strategies to release the pain caused by social exclusion. The excluded individual will give negative evaluations to the person who excluded him/her and will be more likely to act aggressively or antisocially (Buckley et al., 2004). Similarly, aggression is a prominent feature of the Dark Triad (Jones and Neria, 2015). Indeed, recent studies have found that the Dark Triad experiencing rejection, exclusion makes them express much anger and hostility, and even aggression (Baumeister et al., 2000).

Moreover, Gammon et al. (2011) suggest that the Dark Triad traits combined with particular environmental triggering events (specifically ego-threats and social exclusion) lead to patterns of cognitive and affective processing that ultimately result in bullying. While psychopaths may not react to threats against their self-esteem (Jones and Paulhus, 2010; Lämmle et al., 2014), other potential triggering events such as disobedience or challenge from others could still lead them to engage in bullying behavior. Psychopaths have an increased response to provocation and a decreased inhibition of social constraints, which makes highly them extremely prone to anger (Kerig and Stellwagen, 2010). Furthermore, in social exclusion situations, there are both self-threats and physical threats that lead to more aggressive behavior from the excluded individual (Williams et al., 2000; DeWall et al., 2010). According to the cognitive-neoassociation theory (Berkowitz, 1990), when individuals are exposed to negative events in their environment (such as ostracism and exclusion), negative emotions will rise. These negative emotions eventually make the Dark Triad generate school bullying behavior. Empirical studies have shown that high narcissistic individuals exhibit more aggressive behavior following social exclusion (Twenge and Campbell, 2003).

### 1.3. The mediating role of sense of control

Sense of control is a basic human psychological need, and describes a fundamental motivating factor that influences how well one adjusts to life and manages stress. If people lose it, they may experience negative emotions such as depression, anxiety, and anger (Troup and Dewe, 2002). According to social interaction theory (Tedeschi and Felson, 1994), the purpose of aggression is to hurt others or to make them meet the attacker’s three main needs. Therefore, when people experience low sense of control, they are prone to aggression to control the behavior of others to make up for their lacked sense of control. Several empirical studies have found that low sense of control is related to aggressive behavior (Guo et al., 2016; Chen et al., 2018; Ren et al., 2018). For instance, Hall (2006) found that individuals with low dispositional sense of control were more likely to misinterpret neutral facial expressions as angry and engage in more aggressive behavior.

Furthermore, the Dark Triad has low emotional intelligence and limited ability to regulate negative emotions, which results in more bullying behavior (Michels and Schulze, 2021). Manipulating others as one of the most common features of the Dark Triad, they may exhibit distinct emotions and behaviors when their sense of control is deprived, such as bullying (Yuan and Men, 2020). They are more inclined to change their environment or others to gain sense of control rather than adjust themselves. And the latest research also demonstrates that Machiavellians will engage in more relational bullying when their sense of control is briefly stripped away (Wu, 2022).

### 1.4. The serial mediating role of social exclusion and a sense of control

A large amount of research evidence shows that social exclusion is positively associated with negative emotions and alienation, and negatively associated with self-control and sympathy (Leary and Mark, 1990; Twenge et al., 2007; Sun et al., 2017; Jin et al., 2019). Ren et al. (2018) find that ostracized participants have lower sense of control and showed more aggression than included participants. Conversely, aggression is reduced when the ostracized individual’s sense of control is reinforced (Kuehn et al., 2015). Adolescents with psychopathic personality traits are more likely to be excluded by others in interpersonal interactions due to their poorer self-control (Zhang and Zhang, 2014). Additionally, there is a negative relationship between cyber-ostracism and self-control, if individuals experience a frequency of social exclusion, their self-control tends to decrease and they engage in more cyberbullying. Therefore, when the individuals with Dark Triad traits are rejected or excluded, a variety of negative feelings or diminished basic requirements may appear. All of these increase the likelihood of them bullying. As discussed above, social exclusion can lead to a reduced sense of control, which results in bullying behavior.

### 1.5. The present study

In conclusion, the majority of recent studies attach more importance to the relationship between the Dark Triad as a singular factor and adolescent school bullying, while few studies combine the personality traits and the social environment to consider the relationship. Under the basic framework of temporal need-threat model, the purpose of the present study is to bring together cognitive-neoassociation theory and social interaction theory to investigate the effect of the Dark Triad on adolescent school bullying and the mediating mechanisms, so we conducted three studies among Chinese middle school students. In Study 2 and 3, we designed two scenarios to portray everyday social situations which end in either rejection or more amiable outcomes. Our goal was to experimentally induce exclusion and to investigate the subsequent reactions.

The hypotheses to be tested were as follows: (a) the Dark Triad significantly predicts school bullying (*Hypothesis* 1), (b) social exclusion and sense of control would mediate the association between the Dark Triad and school bullying (*Hypothesis* 2), (c) social exclusion and sense of control would play the serial mediating role between the Dark Triad and school bullying (*Hypothesis* 3), (d) Most importantly, adolescents with the Dark Triad traits would experience reduced sense of control and show bullying behavior after being excluded in manipulated conditions (*Hypothesis* 4).

## 2. Study 1

### 2.1. Participants

Study 1 is a model examination at the general level. Through random cluster sampling, we recruited students from a junior middle school in Hubei, China. Our survey was carried out on a class basis, and a total of 571 adolescents (59.4% boys) ranging in age from 12 to 16 (*M*_age_ = 14.53, *SD* = 0.716) participated in this study. Additionally, the following factors were taken into consideration when choosing eligible participants: (I) adolescents who were allowed to participate by their parents, (II) adolescents who agreed to participate. The current study was approved by the Research Ethics Committee of the College of Education and Sports Sciences, Yangtze University. Written consent forms were given to participants and their parents or legal guardians, informing them that their personal information would be kept private and that their answers would only be used for study. The information was gathered by qualified psychology professors or graduate psychology students. To promote truthful reporting, adolescents were given roughly 30 min to complete the confidential questionnaires.

### 2.2. Measures

#### 2.2.1. Dark Triad

The Dark Triad was measured by the Dirty Dozen. The Dirty Dozen is a Dark Triad scale developed by Jonason and Webster (2010), that includes twelve activities that were measured for Machiavellianism, psychopathy, and narcissism, each corresponding to four items. Example activities include “I tend to manipulate others to get my way,” “I tend to want others to admire me,” “and I tend to be callous or insensitive.” A seven-point scale is used (1 = strongly disagree, 7 = strongly agree), calculating the score for each factor and the total score. A high score represents a certain dark trait. The scale has good reliability and validity in the Chinese adolescent population (Geng et al., 2015), with an overall Cronbach’s alpha coefficient of 0.80 and respective Cronbach’s alpha coefficients of 0.78, 0.68, and 0.80 for Machiavellianism, psychopathy, and narcissism. Confirmatory factor analysis (CFA) was conducted to test the extent that the scale structure matched the data, and we found that there was a good fit (*χ*^2^/df = 2.16, *CFI* = 0.98, *TLI* = 0.97, *RMSEA* = 0.05, and *SRMR* = 0.03).

#### 2.2.2. Social exclusion

Social exclusion was measured by the Ostracism Experience Scale for Adolescents (OES-A) measuring adolescents’ experiences of social exclusion (Gilman et al., 2013). The participants were asked to recall their experiences with friends in the recent 6 months, such as “In general, others treat me as if I am invisible” and “In general, others make an effort to get my attention.” The scale is consisted of 11-item on a five-point Likert scale (1 = never, 5 = often). Higher scores indicate higher levels of social exclusion experienced in everyday life. Among Chinese adolescents, this measure showed great validity and reliability (Zhang et al., 2018). In this study, the Cronbach’s alpha was 0.80. CFA was used to test whether the data and the scale structure were compatible, and we found that there was an acceptable fit (*χ*^2^/df = 3.27, *CFI* = 0.96, *TLI* = 0.94, *RMSEA* = 0.06, and *SRMR* = 0.05).

#### 2.2.3. Sense of control

Sense of control was measured with the 12-item self-report questionnaire (Lachman and Weaver, 1998). Example items include “I can do just about anything I really set my mind to.” All items were rated on a seven-point scale ranging from 1 (strongly disagree) to 7 (strongly agree). Mean scores were calculated, with higher scores meaning higher levels of sense of control. In the present study, the Cronbach’s alpha was 0.79. We used CFA to determine whether the scale structure and the data were optimal and we discovered the fit can be accepted (*χ*^2^/df = 4.45, *CFI* = 0.90, *TLI* = 0.86, *RMSEA* = 0.08, and *SRMR* = 0.06).

#### 2.2.4. Traditional bullying

Traditional bullying was measured with the Olweus Bully Questionnaire (Olweus, 1996). The Olweus Bully Questionnaire is a self-report scale that is designed for middle school students, that includes six activities (e.g., “We give others unpleasant nicknames, insult, ridicule or satirize them.”) that were measured for verbal, physical, and relational bullying. The participants were asked to report the frequency of three forms of bullying over the past 6 months on a five-point scale (from 0 = never to 5 = several times a week). We conducted CFA to examine the compatibility of the scale structure with data, and the results showed that there was an acceptable fit (*χ*^2^/df = 4.70, *CFI* = 0.98, *TLI* = 0.94, *RMSEA* = 0.08, and *SRMR* = 0.02). Mean scores were calculated, with higher scores indicating greater severity of traditional bullying. In the present study, the Cronbach’s alpha was 0.75.

#### 2.2.5. Cyberbullying

Cyberbullying is measured with the six-item self-report questionnaire (Lam and Li, 2013). The participants were asked to indicate the frequency with which they had participated in six kinds of cyberbullying such as teasing, making up something, threatening, and calling someone a bad name on the internet during the past 6 months. Specific examples include “How many times did you tease someone using emails, texting, short messages, on a website such as Renren, etc.?” and so on. All items were rated on a seven-point scale ranging from 0 (zero) to 6 (six or more). The Chinese version of the scale has also shown good reliability and validity when used on Chinese adolescents (Hu et al., 2014). In the present study, the Cronbach’s alpha was 0.85. We performed CFA to determine the convenience of the single factor structure with the data and found that there was a good fit (*χ*^2^/df = 2.58, *CFI* = 0.99, *TLI* = 0.98, *RMSEA* = 0.05, and *SRMR* = 0.01).

### 2.3. Statistical analyses

Descriptive statistics and Pearson correlations of the key variables were conducted with SPSS 26.0. And according to previous studies, mediation effects were tested through structural equation modeling using Mplus 8.6 (Wen et al., 2004). Moreover, we adopted bootstrapping with 1,000 replicates to test the hypothesis model.

### 2.4. Results and discussion

Given the possibility of common method bias in the self-report method, we used Harman’s single factor test to examine the common method bias (Podsakoff et al., 2012). The results showed that there are eleven common factors with a characteristic root greater than 1, and the first factor of them is 17.29%, less than the 40% critical standard. That is, the shared method deviation of this study is not serious.

Means, SDs, and bivariate correlations are shown in Table 1. As shown in the table, Machiavellianism (*r* = 0.11, *p* < 0.01) and psychopathy were positively correlated with social exclusion (*r* = 0.18, *p* < 0.001), however, narcissism and social exclusion had no statistical differences. All three dimensions of the Dark Triad were positively associated with four forms of school bullying. Machiavellianism (*r* = −0.14, *p* < 0.001), psychopathy (*r* = −0.24, *p* < 0.001), narcissism (*r* = −0.18, *p* < 0.001), social exclusion (*r* = −0.39, *p* < 0.001), verbal bullying (*r* = −0.27, *p* < 0.001), physical bullying (*r* = −0.28, *p* < 0.001), relational bullying (*r* = −0.19, *p* < 0.001) and cyberbullying (*r* = −0.24, *p* < 0.001) were all negatively associated with sense of control.

**Table 1:** Descriptive statistics and intercorrelations between variables.

Variable	*M*	SD	1	2	3	4	5	6	7	8	9
Machiavellianism	1.90	1.13	1.00								
Psychopathy	1.96	1.11	0.57^***^	1.00							
Narcissism	3.76	1.53	0.27^***^	0.21^***^	1.00						
SE	2.86	0.56	0.11^**^	0.18^***^	−0.02	1.00					
SC	4.53	0.92	−0.14^***^	−0.24^***^	−0.18^***^	−0.39^***^	1.00				
VB	1.12	0.38	0.25^***^	0.24^***^	0.21^***^	0.12^**^	−0.27^***^	1.00			
PB	1.45	0.67	0.23^***^	0.22^***^	0.11^*^	0.16^**^	−0.28^***^	0.45^***^	1.00		
RB	1.19	0.47	0.24^***^	0.21^***^	0.20^***^	0.18^***^	−0.19^***^	0.47^***^	0.41^***^	1.00	
Cyberbullying	0.32	0.73	0.36^***^	0.32^***^	0.17^***^	0.14^***^	−0.24^***^	0.30^***^	0.41^***^	0.34^***^	1.00

SE, social exclusion; SC, sense of control; VB, verbal bullying; PB, physical bullying; RB, relational bullying. ^*^p < 0.05, ^**^p < 0.01, ^***^p < 0.001.

We used independent sample *t*-test to examine the gender differences in the Dark Triad. The results showed that there is no difference between boys and girls in the Dark Triad personality traits (*M*_boys_ = 2.54, *M*_girls_ = 2.52, *t* = 0.27, *p* = 0.79), as do Machiavellianism, psychopathy, and narcissism. To investigate gender differences in bullying among youths, the independent sample t-test was used. Results showed that traditional bullying of boys was significantly higher than that of girls (*M*_boys_ = 3.92, *M*_girls_ = 3.58, *t* = 3.31, *p* < 0.001), with verbal and physical bullying being higher than girls (*t* = 3.74, *p* < 0.001; *t* = 3.14, *p* < 0.05), while there was no significant difference in relational bullying (*t* = 1.27, *p* > 0.05) and cyberbullying (*t* = 0.70, *p* > 0.05).

**Figure 1:**
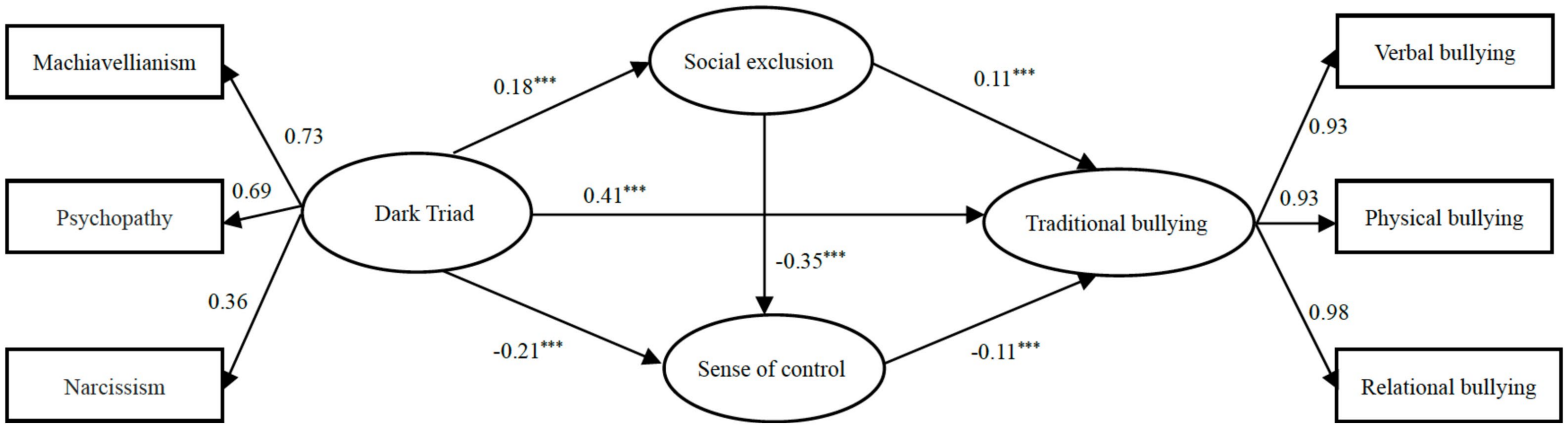
Path model results for traditional bullying with standardized coefficients (*n* = 571). *p < 0.05, **p < 0.01, ***p < 0.001. The same below.

In the beginning, we constructed a measurement model to determine the compatibility of our theoretical model with all data collected within the scope of this study. According to the goodness of fit values (*χ*^2^/df = 2.05, *p* < 0.001; *CFI* = 0.90, *TLI* = 0.89, *RMSEA* = 0.04, and *SRMR* = 0.06), the model had an acceptable fit with the data. We tested the mediation effects of social exclusion and sense of control by following several steps. First, the direct effect of the Dark Triad on adolescent traditional bullying was tested, with the Dark Triad as the predictive variable, gender and age as the controlling variable, and traditional bullying as the outcome variable. The model had a good fit to the data (*χ*^2^/df = 2.86, *p* < 0.001; *CFI* = 0.99, *TLI* = 0.99, *RMSEA* = 0.06, and *SRMR* = 0.03). CFI and TLI are relative fit indices that compare the fit of the hypothesized model to a baseline model. Values close to 1 indicate a good fit. RMSEA is an absolute fit index that estimates the average discrepancy between the observed covariance matrix and the hypothesized model. A value below 0.1 indicates better fit (Steiger, 1990). Finally, SRMR is a measure of the standardized residuals, which should be smaller than 0.08 if the model fits the data well. The results revealed a significant (*β* = 0.45, *p* < 0.001) direct path from the Dark Triad to traditional bullying. Then we added two mediation variables, social exclusion and sense of control, to the model to build a serial mediation model. The mediation model fit the data well (*χ*^2^/df = 3.72, *p* < 0.001; *CFI* = 0.98, *TLI* = 0.97, *RMSEA* = 0.07, and *SRMR* = 0.03). The results are presented in Figure 1, which indicates that all paths were statistically significant.

In order to effectively control measurement error, this study uses structural equation model to examine multiple mediating effects. Bootstrapping analyses was used to test the relationship between each path and if the 95% confidence interval did not include 0, then the indirect effect was significant. The results indicated that social exclusion (*95% CI*: 0.008, 0.042) and sense of control (*95% CI*: 0.012, 0.044) partially mediated the relationship between the Dark Triad and traditional bullying, and that social exclusion-sense of control (*95% CI*: 0.003, 0.015) mediated the serial.

**Figure 2:**
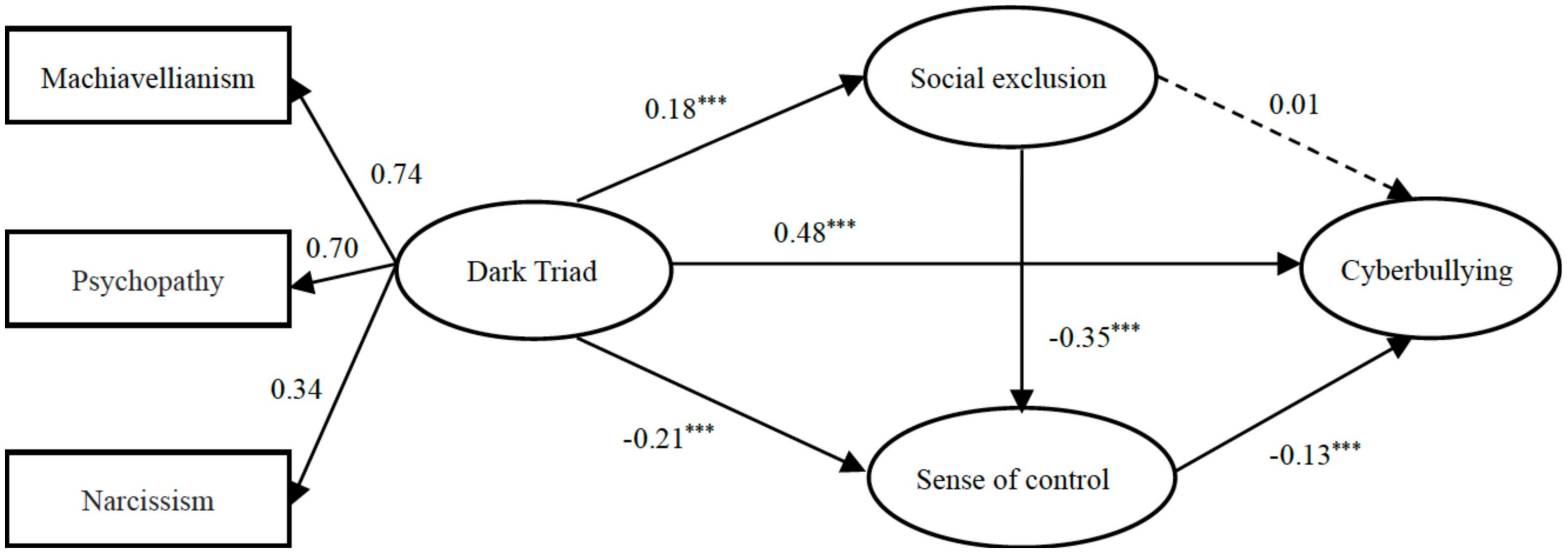
Path model results for cyberbullying with standardized coefficients (*n* = 571).

Perform the same steps as above, through the measurement models we got a not bad model fit (*χ*^2^/df = 2.04, *p* < 0.001; *CFI* = 0.91, *TLI* = 0.90, *RMSEA* = 0.04, and *SRMR* = 0.06). And then, after controlling gender and age, we tested the direct effect of the Dark Triad on adolescent cyberbullying, and the path coefficient was found to be significant (*β* = 0.67, *p* < 0.001). Next, the mediating variables, social exclusion and sense of control, were added to the model to obtain the path model. The mediation model fit the data well [*χ*^2^/df = 4.70 (*p* < 0.001), *CFI* = 0.96, *TLI* = 0.89, *RMSEA* = 0.08, and *SRMR* = 0.03]. The results are presented in Figure 2, which indicates that one path was not statistically significant.

The results of the mediating analysis showed that sense of control (*95% CI*: 0.086, 0.694) partially mediated the relationship between the Dark Triad and traditional bullying, and that social exclusion-sense of control (*95% CI*: 0.021, 0.244) mediated the serial, but social exclusion did not (*95% CI*: −0.2, 0.197).

Although our study 1 did not directly conduct a power analysis, we can discuss our results based on the effect size estimates from similar studies. In our study, we tested specific research hypotheses through structural equation modeling analysis. According to Wolf et al. (2013), our sample size may be able to detect medium effect. However, it should be noted that the detected effect size may be influenced by various factors such as sample characteristics, reliability of measurement tools and model fitness (Muthén and Muthén, 2002). Therefore, we should interpret the effect sizes in our results with caution and discuss them in the context of relevant literature and practical background.

Taken as a whole, Study 1 provided evidence for our hypotheses 1, 2 and 3 using a correlational design with self-reported measures. However, Study 1 was a correlational study that could not verify the bullying behavior of the Dark Triad being caused by whether they have been excluded in real situations. In addition, participants’ responses to the school bullying measure may be subject to social desirability bias, such that they may tell lower levels of bullying than they factually experience. To address these limitations, in Study 2 and 3 we sought to manipulate participants’ social relationship to examine its casual effect on school bullying and provide additional evidence for the mediation model.

## 3. Study 2

In this study, we used a 2(the Dark Triad traits: high Dark Triad traits vs. low Dark Triad traits) *2(social relationships: exclusion vs. acceptance) between-group experimental design. We further manipulated participants’ social relationships separately by randomly assigning participants to one of the two groups (“social exclusion” or “social acceptance”). To verify the effectiveness of the social exclusion material manipulation, 44 adolescents were recruited to test the effect of the manipulation, using two items for assessment (“I feel rejected” and “I feel excluded,” 1 = “strongly disagree” to 7 = “strongly agree”) (Buckley et al., 2004; Ding and Gong, 2016). The result of the paired samples t-test showed that the manipulation was valid (*t* = 6.63, *p* < 0.001).

### 3.1. Participants

An *a priori* power analysis (the estimated effect size of *f* = 0.4, *α* = 0.05, power = 0.80) suggested a required sample size of *N* = 73. The participants in this study included 160 junior high school students in China. The Dark Triad scores were ranked from high to low, with the top 27% being the high group and the bottom 27% being the low group. The final effective number of respondents was 88, with 44 in both the high and low groups. An independent samples *t*-test was conducted on the Dark Triad scores of the two groups, and the results showed that the high group (*M* = 50.14, *SD* = 6.70) was significantly higher than the low group (*M* = 21.20, *SD* = 4.52), *t*(86) = 23.76, *p* < 0.001.

### 3.2. Procedure and materials

After providing informed consent, participants first reported their personal information and filled out the Dirty Dozen scale. Then, they were randomly assigned to one of two conditions: “social exclusion” (*n* = 55), “social acceptance” (*n* = 33). Afterwards, they needed to complete two manipulation tests, measurement scale of sense of control and bullying. We used the same measurements as in Study 1 to assess participants’ sense of control, traditional bullying and cyberbullying. However, some minor changes were made to the cyberbullying scale (we removed “I have ever” from all six items), as this expression was not appropriate for this scenario study. A validation factor analysis of the questionnaire showed that the model fitted well: *χ*^2^/df = 1.31, *CFI* = 0.99, *TLI* = 0.99, *RMSEA* = 0.06, *SRMR* = 0.03. The Cronbach’s α of the six items of was 0.90.

### 3.3. Manipulation of social relationships

We asked the participants to read the material carefully, to make themselves characters in the story by thinking and feeling as if they were actually experiencing the situation, and to complete the subsequent questions. The manipulation material was adapted from Breen and Kashdan (2011). The premise of the story was the same for both groups, in which the participant and new classmates agreed to form a group to work together on a task set by the teacher. After their dinner, the social exclusion group was told that his/her new classmates had formed a group with another person, excluding him/her, whereas the social acceptance group was told that he/she had formed a group with his/her new classmates. Next, participants responded to two manipulation check questions (same as above).

### 3.4. Analytic plan

A two-way analysis of variance (ANOVA) was conducted in SPSS 26.0 to test the difference in sense of control and school bullying across the four conditions. To test Hypotheses 3, we first created two dummy variables for the Dark Triad and social relationships (“high Dark Triad” condition was coded as 0, “low Dark Triad” condition was coded as 1; “social acceptance” condition was coded as 0, “social exclusion” was coded as 1) and then conducted the analysis of variance.

### 3.5. Results and discussion

The results of the ANOVA to test the manipulation effect of exclusion showed a significant difference between the two groups. As intended, the participants in the exclusion group had a higher sense of rejection than the acceptance group, *F*(1, 86) = 55.61, *p* < 0.001; the exclusion group also had a higher sense of exclusion than the acceptance group, *F*(1, 86) = 30.32, *p* < 0.001, indicating that the material was effective in initiating exclusion.

A one-way ANOVA on sense of control indicated a significant difference across the two social relationships, *F*(1, 86) = 20.50, *p* < 0.001. The result showed that participants in the “social exclusion” condition (*M* = 3.79, *SD* = 0.93) felt lower sense of control than the “social acceptance” condition (*M* = 4.62, *SD* = 0.64).

**Figure 3:**
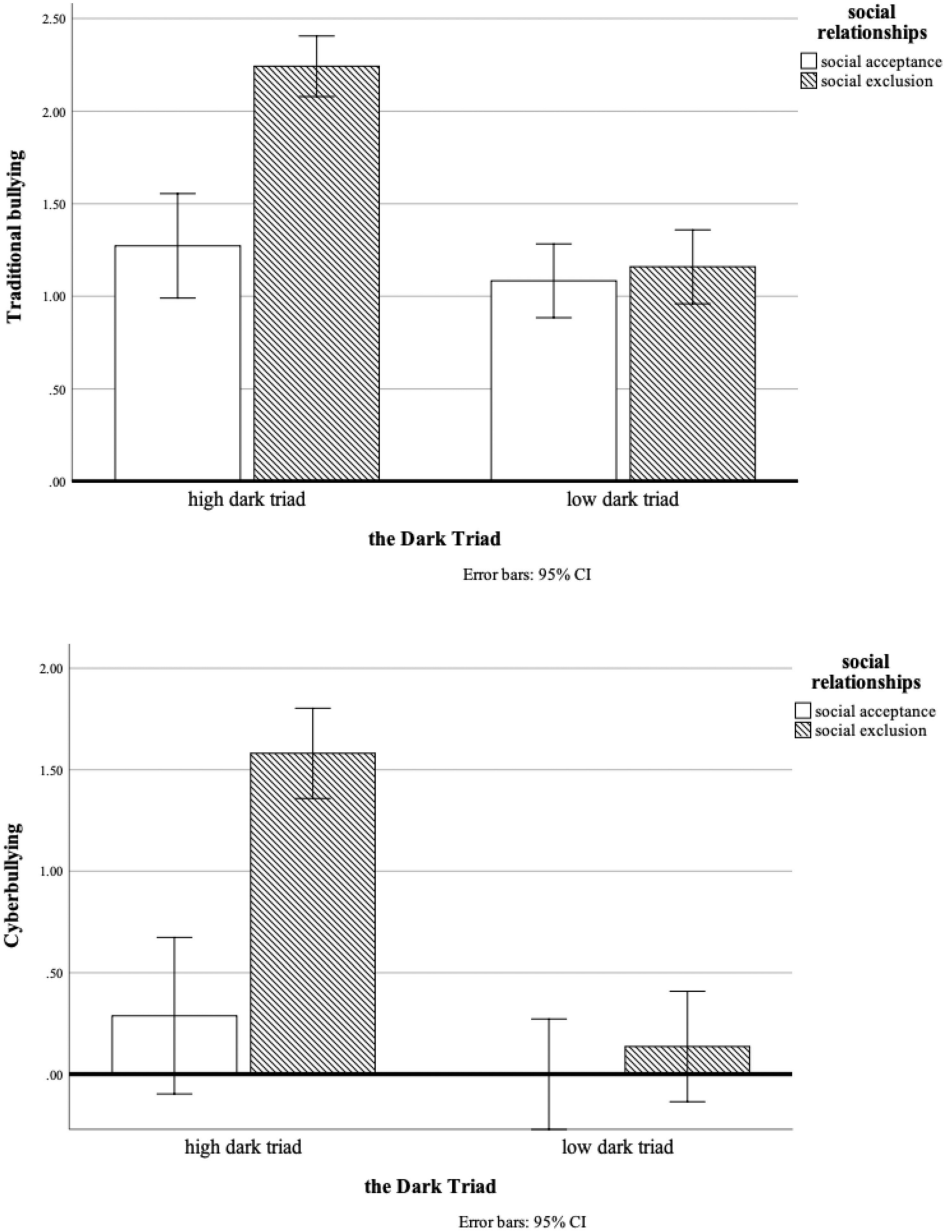
Results of the interaction between the dark triad and social relationships under the manipulated condition.

We first conducted a 2 (high and low Dark Triad) × 2 (social relationships) ANOVA on the data, with traditional bullying as the dependent variable. The results of the one-way between-group ANOVA (see Table 2) showed a significant main effect of Dark Triad [*F*(1, 84) = 34.41, *p* < 0.001], a significant main effect of social relationships [*F*(1, 84) = 23.22, *p* < 0.001], and a significant interaction effect between the two (*F*(1, 84) = 16.97, *p* < 0.001) (See Figure 3). To examine the impact of the Dark Triad on traditional bullying by different social relationships, we continued with a simple effect analysis. In the social exclusion scenario, the main effect of the Dark Triad was significant [*F*(1, 84) = 69.80, *p* < 0.001], with the high Dark Triad having more traditional bullying compared to the low Dark Triad. In the social acceptance scenario, the main effect of the Dark Triad was not significant, *F*(1, 84) = 1.19, *p* > 0.05.

**Table 2:** ANOVA table for the Dark Triad, social relationships on traditional bullying.

Source of variation	Sum of squares	df	mean square	*F*	*p*	η^2^
the Dark Triad	7.64	1	7.64	34.41***	<0.001	0.29
Social relationships	5.15	1	5.15	23.22***	<0.001	0.22
the Dark Triad*Social relationships	3.77	1	3.77	16.97***	<0.001	0.17
Error	18.64	84	0.22			

^***^p < 0.001.

Then, we performed the same steps with cyberbullying as the dependent variable. The results of the one-way between-group ANOVA (see Table 3) showed a significant main effect of Dark Triad [*F*(1, 84) = 34.29, *p* < 0.001], a significant main effect of social relationships [*F*(1, 84) = 23.34, *p* < 0.001], and a significant interaction effect between the two [*F*(1, 84) = 15.28, *p* < 0.001] (See Figure 3). The results of the simple effect analysis show that the main effect of the Dark Triad was also significant [*F*(1, 84) = 66.75, *p* < 0.001] in the social exclusion condition, suggesting that the high Dark Triad also generates more cyberbullying than the low Dark Triad. In the social acceptance condition, the main effect of the Dark Triad was not significant, *F*(1, 84) = 1.47, *p* > 0.05.

**Table 3:** ANOVA table for the Dark Triad, social relationships on cyberbullying.

Source of variation	Sum of squares	df	Mean square	*F*	*p*	η^2^
The Dark Triad	14.15	1	14.15	34.29***	<0.001	0.29
Social relationships	9.63	1	9.63	23.34***	<0.001	0.22
The Dark Triad*Social relationships	6.31	1	6.31	15.28***	<0.001	0.15
Error	34.66	84	0.41			

^***^p < 0.001.

The results of this study confirm previous hypothesis 4 in that social exclusion was the main trigger of bullying in adolescents with high Dark Triad and that social exclusion also contributed to their reduced sense of control. When adolescents felt that they were lacking a means of control, no matter whether conditional or characteristic, they made more bullying choices. Reciprocally, when high Dark Triad traits adolescents are in a social acceptance situation, they have a relatively balanced sense of control. Only by priming social exclusion did they induce subsequent bullying. These results support the claim that an individuals’ bullying is partly influenced by personality traits and undesirable environment and are consistent with another research using this paradigm (Ferguson and Dyck, 2012; Chen et al., 2018).

The finding that social exclusion materials influence high dark traits adolescent’s bullying in this experiment should be taken into further analysis. Although it was not the main cause of the bullying, the influence of social exclusion situation on an adolescent’s bullying, combined with a low sense of personal control, indicates a feasible model for understanding why adolescents may act out violently.

## 4. Study 3

We performed exactly the same procedure as in Study 2, except for the social relationships manipulation material—we changed the offline situation to an online one to explore whether online social exclusion could lead to the same results. Cyber-ostracism is an extension of reality social exclusion in a cyber context (Schneider et al., 2017), and many studies have shown that cyber-ostracism is positively related to both traditional aggression and online aggressive behavior (Dewall et al., 2009; Jin et al., 2019). Therefore, we will use a different scenario to further test our hypothesis 4.

### 4.1. Participants

The participants in this study included 187 senior high school students in China. The Dark Triad is grouped in the same way as Study 2. The final effective number of respondents was 102, with 51 in both the high and low groups. An independent samples *t*-test was conducted on the Dark Triad scores of the two groups, and the results showed that the high group (*M* = 46.75, *SD* = 11.17) was significantly higher than the low group (*M* = 21.94, *SD* = 4.01), *t*(100) = 14.93, *p* < 0.001.

### 4.2. Procedure and materials

After providing informed consent, participants first reported their personal information. Then, they were randomly assigned to one of two conditions: “social exclusion” (*n* = 49), “social acceptance” (*n* = 53). They also completed measures of manipulation checks, sense of control, traditional bullying, and cyberbullying.

### 4.3. Manipulation of social relationships

The manipulation method was adapted from the contextual material in the study by Wan et al. (2014). The participants were first asked to read a story and then put themselves into the role of the main character in the story. Specifically, the participants needed to get help from several unfamiliar schoolmates and took the initiative to add them as friends on social media platforms, and after 3 days, they received different dating feedback. The social exclusion group received feedback that all three people rejected the request, while the social acceptance group received feedback that all three people accepted the request.

### 4.4. Results and discussion

The results of the ANOVA test to determine whether exclusion had a manipulative effect revealed a significant difference between the two groups. According to plan, participants in the exclusion group felt more rejected than those in the acceptance group, *F*(1, 100) = 71.49, *p* < 0.001, and more excluded than those in the acceptance group, *F*(1, 100) = 31.64, *p* < 0.001, showing that the material was successful in causing exclusion.

**Figure 4:**
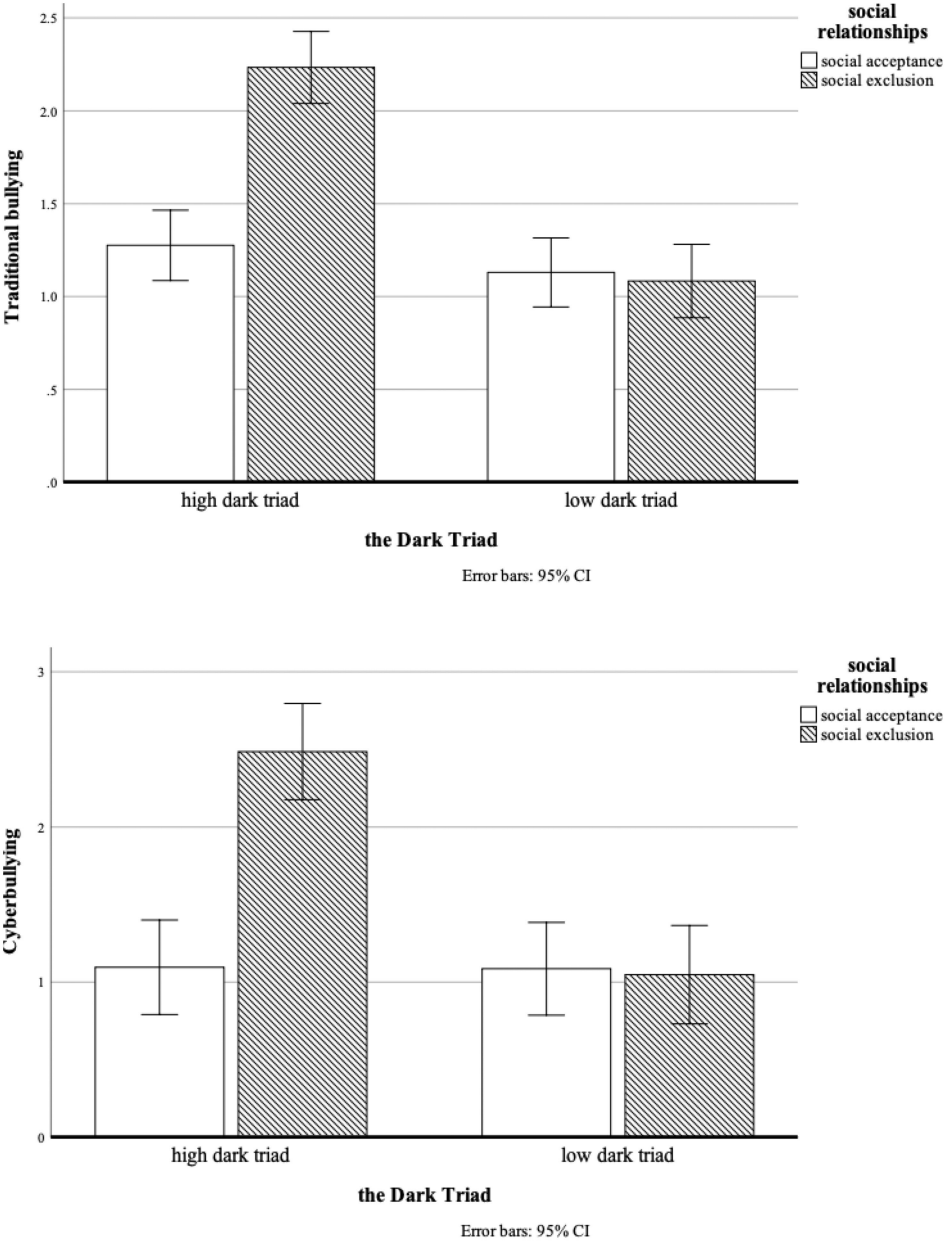
Results of interaction effects.

A one-way ANOVA on sense of control revealed a significant difference between the two social relationships, *F*(1, 100) = 15.03, *p* < 0.001. Participants in the “social exclusion” condition (*M* = 3.96, *SD* = 0.89) felt less sense of control than those in the “social acceptance” condition (*M* = 4.58, *SD* = 0.172), according to the results. With the traditional bullying as the dependent variable, the results of the one-way between-group ANOVA showed a significant main effect of Dark Triad [*F*(1, 98) = 44.96, *p* < 0.001], a significant main effect of social relationships [*F*(1, 98) = 22.24, *p* < 0.001], and a significant interaction effect between the two [*F*(1, 98) = 26.98, *p* < 0.001] (see Figure 4). The results of the simple effects analysis indicated that in the social exclusion scenario, the main effect of the Dark Triad was significant [*F*(1, 98) = 68.13, *p* < 0.001], and in the social acceptance scenario, the main effect of the Dark Triad was not significant, *F*(1, 98) = 1.19, *p* > 0.05. Then, using cyberbullying as the dependent variable, we repeated the process. A significant main effect of the Dark Triad [*F*(1, 98) = 21.82, *p* < 0.001], a significant main effect of social relationships [*F*(1, 98) = 19.05, *p* < 0.001], and a significant interaction effect between the two [*F*(1, 98) = 21.24, *p* < 0.001] (see Figure 4) have all been found in the one-way between-group ANOVA results. The results of the simple effect analysis indicated that the main effect of the Dark Triad was significant in the social exclusion condition [*F*(1, 98) = 41.44, *p* < 0.001], and the Dark Triad’s main effect was insignificant in the social acceptance condition, *F*(1, 84) = 0.002, *p* > 0.05. All the results are the same as in Study 2.

Thus, through two experimental studies that manipulated social relationships in the forms of exclusion and acceptance, Study 2 and 3 further supported our hypotheses 4 when testing the casual effect of the Dark Triad and social exclusion on adolescent bullying and its underlying mechanisms.

## 5. General discussion

In the current study, we aimed to analyze the impact of the Dark Triad on different types of bullying (traditional and cyberbullying) in adolescents. More specifically, we aimed to determine whether social exclusion and sense of control were associated with engagement in traditional bullying or cyberbullying and if this engagement was associated with being excluded and reduced sense of control. We propose that social exclusion situation and a low sense of control both are core factors in inducing adolescent bullying intent. Specifically, adolescents become more aggressive in particular when they feel their circumstances are beyond their control, which lessens the psychological stress of their distress and anxiety (Hall, 2006; Sullivan et al., 2010).

The results showed that boys scored higher than girls in physical and verbal bullying, indicating that boys engaged in more physical and verbal bullying, which was consistent with previous findings (Beran and Tutty, 2002; Wang et al., 2009). This direct form of bullying is more common among boys, because they are more extroverted and impulsive than girls. On the other hand, Machiavellianism and psychopathy were positively correlated with social exclusion, but narcissism was not. Machiavellianism is too concerned with self-interest to trust others (Rogoza and Cieciuch, 2018). Psychopathy is impulsive and not honest enough (Sehar and Fatima, 2016). Some of the bad traits they possess may lead to them being unacceptable to the team and excluded by their mates. In fact, narcissism is relatively the ‘brighter’ trait of the Dark Triad, due to the fact that narcissists are charismatic, self-motivated and have higher emotional intelligence (Scavone, 2017), so it is less likely to be excluded than the other two traits. In line with our hypothesis 1, we found that the three dimensions of the Dark Triad are positively related to four forms of bullying, indicating that individuals with high Dark Triad traits are more likely to commit bullying. Even in China, which is founded on a Confucian culture, Machiavellianism and psychopathy had greater predictive effect values for bullying than narcissism, and this is in accordance with previous studies conducted in other nations (Kish-Gephart et al., 2010; Davis et al., 2022). Machiavellianism and psychopathy are both callous, apathetic, and disregard for morality (Qin and Xu, 2013), so individuals with these high traits are likely to display bullying and aggressive behavior, whether for manipulative, thrill-seeking or revengeful purposes (Zhu and Jin, 2021). The exploitativeness of narcissism can also make it a predictor of bullying, for example, Ang et al. (2010) reported a significant correlation.

Moreover, structural equation model analysis indicated that the mediating effect of social exclusion and sense of control was significant, that is, the Dark Triad can not only directly predict bullying but also indirectly affect bullying through social exclusion and sense of control. The temporal need-threat model suggests that social exclusion threatens individuals’ basic needs such as sense of belonging and control, and that individuals may resort to bullying behavior to release their pain (Williams, 2009). In this study, social exclusion is negatively associated with sense of control and positively associated with school bullying, both of which are consistent with previous research (Hwang and Mattila, 2019; Mazzone et al., 2021; Tunel and Kavak, 2022). According to social interaction theory (Tedeschi and Felson, 1994), the Dark Triad experience a lower sense of control, for which they would bully others in order to compensate for their sense of control. It is worth noting that in Study 1 we found that social exclusion was not a predictor of cyberbullying. We believe that the problem may be caused by several reasons. First, participants in this study were middle school students in the age range of 12–16 years old. Students in this age range are busy with study and have little access to the internet during their school days, so they cannot bully others online when they are excluded in school. Second, when they are ostracized in school, they are more likely to choose other forms of bullying rather than cyberbullying because this offline bullying is more direct and quicker than cyberbullying and does not need to wait until they can use the internet on holiday. However, if they face a depletion of natural resources due to chronic exclusion, such as a reduced sense of control, individuals will respond with a range of behaviors (Williams, 2009). At this moment, they are more likely to engage in cyberbullying. What’s more, in Study 2 and 3 we can know that the Dark Triad shows more cyberbullying after social exclusion.

Our findings also indicated that social exclusion could explain why the Dark Triad predicted school bullying. This is a novel finding because it went beyond previous findings that the interaction of the Dark Triad and social exclusion leads to more bullying in school (Gammon et al., 2011). In our studies, both the experience of social exclusion and the experimental manipulation of social exclusion significantly affected participants’ sense of control and school bullying. Especially after suffering social exclusion, adolescents with high Dark Triad would develop more bullying behavior. The results reliable backup the personality process model put forth by Gammon et al., which states that bullying behavior can be brought on by the Dark Triad in conjunction with particular triggering situations (such as social exclusion). Social exclusion is a threat to the need to belong and the need for relationships, which prevents individuals from meeting their basic needs and may therefore lead to feelings of frustration, eventually inducing bullying. The excluded individuals feel a lack of control and much of their behavior is an effort to re-establish their sense of control. One study investigating chronically excluded individuals showed that they engage in attention-seeking behaviors (making loudness, fighting, etc.) in order to receive feedback (Williams, 2009). Thus, they may use bullying behavior as a way to get the attention of others in order to re-establish a sense of control. After experiencing exclusion and losing sense of control, individuals with the Dark Triad do not choose to adjust themselves to their environment but tend to change their environment or others to regain sense of control, meaning that all forms of bullying are possible. Additionally, we might surmise that bullying may happen when Dark Triad adolescents attempt to manipulate others but are rejected or they are queried by others. Besides social exclusion, the Dark Triad could even contribute to bullying at school from any other negative circumstances that put the ego in threat. We believe the current studies can extend these theories to understand the Dark Triad bullying, and that these models may also be applicable to other personality traits or adults. When the Dark Triad (or neuroticism) is confronted with situations of social exclusion or rejection, their cognitive and emotional structures are altered, and this can be a potential risk for triggering bullying intent (Boyes and French, 2009). This potential risk increases when their sense of control is lowered.

## 6. Implications, limitations, and future directions

Although studies have confirmed the separate predictive effects of the Dark Triad and social exclusion on school bullying, there is limited evidence combining the two and their underlying mechanisms. We found that social exclusion does lead to bullying behavior, and that this effect was most prominent in people who had high levels of the Dark Triad. This study extends the research on the factors influencing school bullying in adolescents by exploring the relationships and mechanisms between the Dark Triad and school bullying from a motivational perspective, based on the temporal need-threat model, the personality process model and so on. The findings contribute to the increasing amount of research on the topic of bullying in schools among teenagers and offer a solid empirical foundation and intervention strategies to avoid bullying so that the issue can be addressed logically and scientifically. By raising awareness of good friendships, improving sense of control, and encouraging prosocial rather than aggressive behavior among adolescents’ mental health practices, the study will help reduce bullying in schools. First, as the parent–child relationship is the foundation for friendship formation and development, this suggests that parents should concentrate on creating a loving family environment for their children, thereby promoting the establishment and development of good friendships. Additionally, in an environment of close friendship, people learn from each other and share experiences, contributing to the development of optimistic attitudes and reducing poor coping strategies. Then, bullying becomes less common as a result of this. Second, we can improve the Dark Triad’s sense of control by self-affirmation. Research has found that self-affirmations are effective in enhancing psychological quality and positive social attitudes, especially for disadvantaged groups (Sherman et al., 2000).

The present research also has some limitations that should be noted, which need to be further improved in future studies. Firstly, although the study used two experimental designs to infer the causal relationships between variables, further investigation and verification can be conducted in the future by combining experimental and longitudinal studies to reveal the mechanisms of variables in greater depth. For example, the developmental trajectory of bullying behavior in individuals with the Dark Triad who are chronically socially excluded. Secondly, data was collected only through self-report measures. Self-reporting may be subject to more bias (e.g., socially desirable responses) and participants may report less school bullying. Even in the experimental cases of Study 2 and 3, our measure of school bullying was a self-report method that did not better avoid this effect. Reports from multiple informants (e.g., parents, teachers, and peers) should be considered in future research. Thirdly, it is one of also our limitations for using the Dirty Dozen to measure the Dark Triad personality traits. Some studies have shown that Dirty Dozen did not evaluate important variations in interpersonal confrontation and inhibition, so researchers suggest that caution should be exercised when using dependence on Dirty Dozen as a measure of mental illness (Miller et al., 2012). In addition, due to the brevity of the scale, it cannot capture all aspects included in the higher-order dimensions of the Dark Triad personality. Therefore, better measurement methods such as Short Dark Tetrad (Paulhus et al., 2020) should be adopted or using mature scales to measure each personality trait separately in future. Fourthly, the sample was limited to adolescents drawn from three middle schools in China. Therefore, care should be taken when extrapolating the findings to other cultures. The findings of the current study must also be expanded to include a more representative sample of Chinese adolescents and adolescents from different cultural backgrounds for a wider test. Finally, there are two developmentally different pathways for rejected youth, one characterized by social withdrawal and shyness, and the other associated with aggressive and inappropriate behavior (McDougall et al., 2001). In our research, we only explored the second scenario. In the future, we need to consider both pathways in order to make a comprehensive analysis.

## 7. Conclusion

The Dark Triad personality traits can be positively associated with adolescent school bullying, and social exclusion and sense of control play a serial mediating role between the Dark Triad and among adolescents. We investigated the types of social relationships and how they influence school bullying in individuals with the Dark Triad through two experimental studies. In general, social exclusion increases school bullying, a result that can be attributed to a decreased sense of control.

## Data availability statement

The raw data supporting the conclusions of this article will be made available by the authors, without undue reservation.

## Ethics statement

The studies involving human participants were reviewed and approved by the Research Ethics Committee of the College of Education and Sports Sciences, Yangtze University. Written informed consent to participate in this study was provided by the participants’ legal guardian/next of kin. Written informed consent was obtained from the individual(s), and minor(s)’ legal guardian/next of kin, for the publication of any potentially identifiable images or data included in this article.

## Author contributions

XG designed the study. SR, BG, ZH, and ZW collected and analyzed the data. YH drafted the manuscript. XG, XJ, and YH revised the manuscript. All authors contributed to the article and approved the submitted version.

## Funding

This research was supported by Youth project of Science and Technology Research Plan of Department of Education of Hubei Province in 2020 (Q20201306), the Social Science Fund Project of Yangtze University in 2022 (2022csz03), the Faculty Scientific Fund Project of the College of Education and Sports Sciences of Yangtze University in 2022 (2022JTB01), and the key projects of education science plan of Hubei Province in 2022: Study on the influencing factors and intervention mechanism of non-suicidal self injurious behaviors in adolescents (2022GA030).

## Conflict of interest

The authors declare that the research was conducted in the absence of any commercial or financial relationships that could be construed as a potential conflict of interest.

## Publisher’s note

All claims expressed in this article are solely those of the authors and do not necessarily represent those of their affiliated organizations, or those of the publisher, the editors and the reviewers. Any product that may be evaluated in this article, or claim that may be made by its manufacturer, is not guaranteed or endorsed by the publisher.
